# A Rare Case of Motor Neuropathy Secondary to Non-Hodgkin Lymphoma

**DOI:** 10.7759/cureus.114023

**Published:** 2026-08-05

**Authors:** Daniel Correa Lopez, Mauricio Estrada, Ricardo Uribe González, Ricardo Cardona, Camila Velez

**Affiliations:** 1 Radiology, CES University School of Medicine, Medellín, COL; 2 Radiology, CES University School of Medicine, Hospital Pablo Tobón Uribe, Medellín, COL; 3 Radiology, Hospital Pablo Tobón Uribe, Medellín, COL; 4 Pathology, Hospital Pablo Tobón Uribe, Medellín, COL; 5 General Medicine, Universidad Pontificia Bolivariana, Medellín, COL

**Keywords:** magnetic resonance imaging, motor mononeuropathy, neurolymphomatosis, non-hodgkin lymphoma, paraneoplastic syndromes, peripheral motor neuropathy

## Abstract

Neurological syndromes associated with lymphomas are uncommon. These syndromes occur mainly in Hodgkin lymphoma and primarily involve the central nervous system. Neurological manifestations in the peripheral nervous system and motor neuropathies are rare and add an extra layer of diagnostic confusion. We describe the case of a 70-year-old man with a history of right foot drop, who arrived at the emergency department after sustaining polytrauma. During initial evaluation, imaging studies incidentally revealed a retroperitoneal mass, which further studies diagnosed as an aggressive B-cell lymphoma with MYC mutation. After multidisciplinary evaluation, clinical findings supported the diagnosis of right peroneal nerve motor mononeuropathy as a paraneoplastic syndrome secondary to lymphoma. We describe the imaging findings, diagnostic workup, and clinical course that enabled recognition of this atypical association.

## Introduction

Neurological manifestations in patients with non-Hodgkin lymphoma (NHL) are varied and may include central nervous system involvement, peripheral neuropathies, and dermatomyositis, among others. Motor neuropathy associated with lymphoma represents a diagnostic and therapeutic challenge due to its nonspecific presentation and the need to differentiate it from other inflammatory or metabolic neuropathies [1].

Differentiating paraneoplastic neuropathy from neurolymphomatosis (NL) is essential. Paraneoplastic neuropathy represents an immune-mediated response secondary to cancer, whereas NL is the direct infiltration of malignant cells into nerve tissue [1-3]. Paraneoplastic neuropathy is caused by an autoimmune response against antigens shared between the tumor cells and neurons, mediated by onconeural antibodies [1,3]. NL represents mechanical and metabolic damage resulting from the proliferation of malignant cells that invade the intra-, extra-, or parafascicular compartments of the nerves [2].

Grisold et al. described peripheral neuropathies associated with lymphomas, emphasizing their heterogeneous presentation and the need for early recognition to enable appropriate management [2,3]. In paraneoplastic syndromes, immunologic mediators contribute to the pathophysiology; the detection of onconeural antibodies in serum and cerebrospinal fluid samples from affected patients supports the association. The mentioned neuropathies have distinct diagnostic criteria that, if recognized, allow for a more accurate diagnosis and help distinguish them from other neuropathies [1,4,5].

From a clinical point of view, paraneoplastic neuropathy generally presents as a subacute sensory neuropathy characterized by progressive loss of pain and vibration sensation [1,3]. NL has a broader range of clinical manifestations that include mononeuropathies or plexopathies, presenting with intense and asymmetric pain [2].

Imaging is key to diagnosing neurologic manifestations and characterizing paraneoplastic neurological syndromes (PNS), highlighting the utility of ultrasound, magnetic resonance imaging (MRI), computed tomography (CT), and positron emission tomography (PET) [4]. A well-founded clinical suspicion, based on prior nerve conduction studies and electromyography (EMG), invariably complements these studies.

On imaging studies, paraneoplastic neuropathy on MRI may show subtle findings such as a smooth, thin enhancement of the nerves [1]. In contrast, neurolymphomatosis is typically identified by focal or diffuse nerve thickening and intense glucose uptake on PET/CT, reflecting the high metabolic activity of the infiltrating lymphoma [1,2]. The diagnosis of paraneoplastic syndromes requires the identification of specific antibodies (such as anti-Hu or anti-CRMP5) [1,3]. Finally, to confirm neurolymphomatosis, the gold standard is tissue biopsy [2].

We describe the case of a 70-year-old patient with NHL who developed progressive motor neuropathy. This article reinforces the need for early recognition of lymphoma-associated neuropathies and the consideration of a multidisciplinary approach to their diagnosis and management.

## Case presentation

A 70-year-old man was under initial follow-up by the physiatry service for a 6-month progressive history of right foot drop accompanied by ipsilateral foot paresthesias. The weakness was steadily progressive, transitioning from Medical Research Council (MRC) grade 4/5 (++) to grade 2/5 (+) over three months, eventually resulting in a complete inability to dorsiflex the ankle. Notably, the patient did not experience any associated localized or radicular pain during this period. Initial EMG findings were consistent with denervation of the right peroneal nerve at the level of the knee, sparing the radicular level.

**Figure 1:**
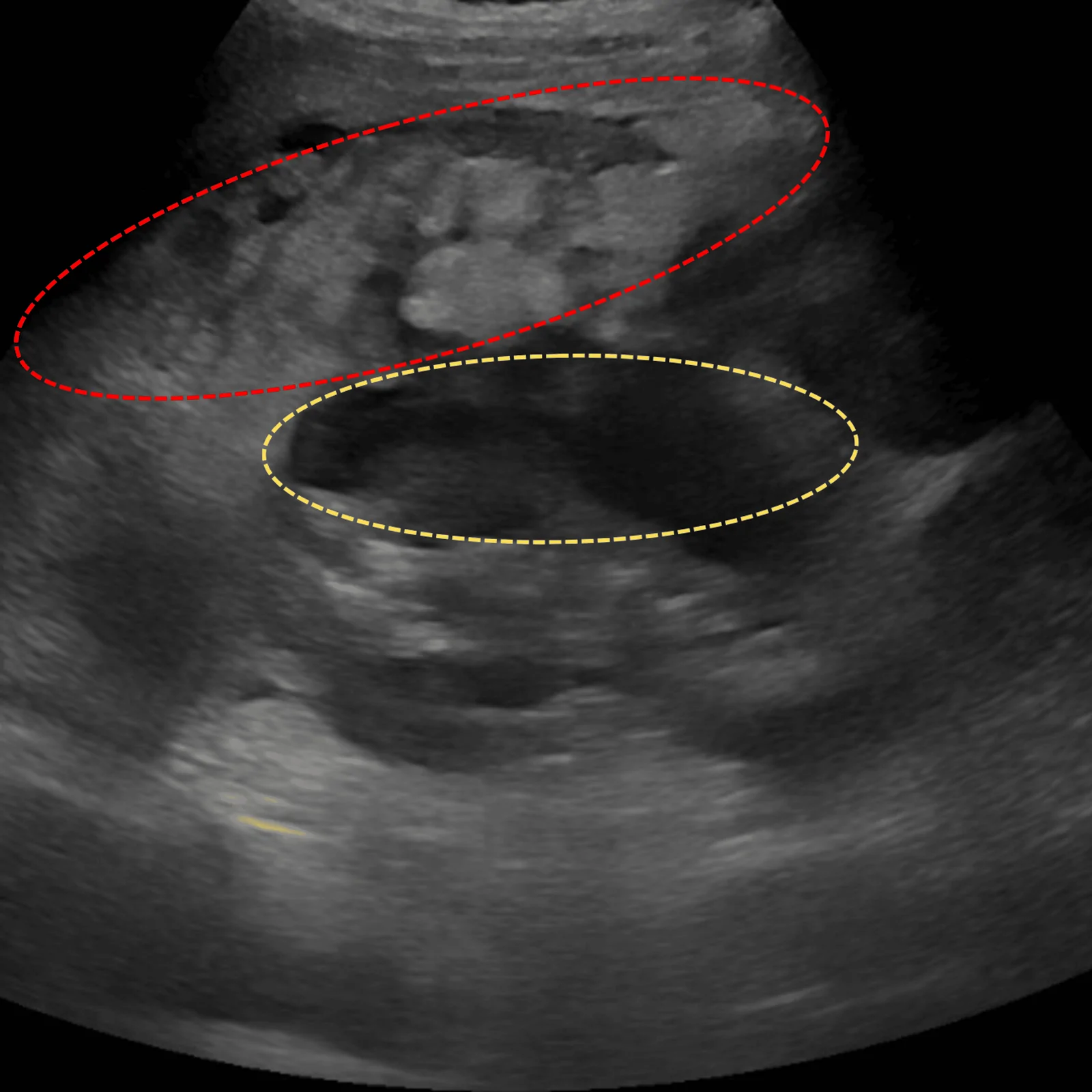
Abdominal ultrasound An echogenic heterogeneous mass with a polypoid and infiltrative appearance was identified occupying the right anterior pararenal space (red lines). Adjacent to the lesion, a subcapsular renal hematoma with an estimated volume of approximately 80 cc was observed (yellow lines). A contrast-enhanced abdominal computed tomography (CT) scan was recommended for further characterization.

The patient was admitted to the emergency department several months later due to polytrauma, presenting with blunt abdominal and pelvic trauma. Initial evaluation included an abdominal ultrasound, which incidentally identified a mass in the pararenal space (Figure 1).

**Figure 2:**
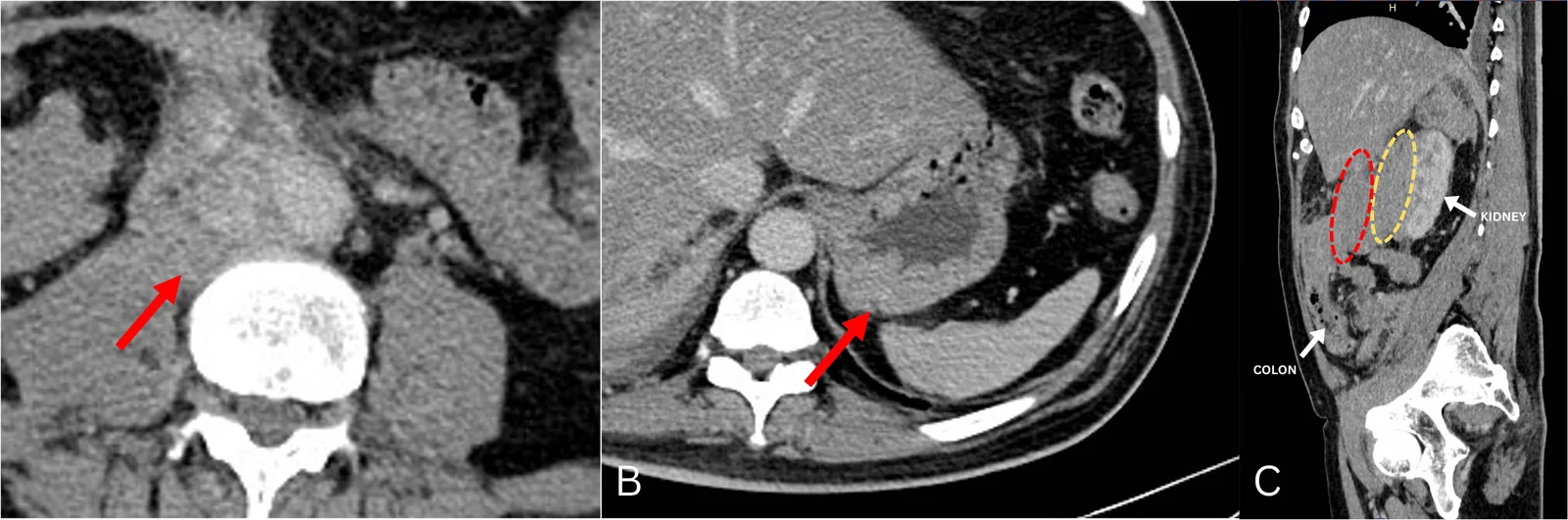
Contrast-enhanced abdominal CT scan A: Retroperitoneal lymphadenopathy with conglomerate formation; B: Asymmetric mural thickening of the greater curvature of the stomach; C: Oblique sagittal image demonstrating a subcapsular renal hematoma (yellow lines) and a heterogeneous hypoenhancing anterior pararenal mass (red lines) extending to the hepatic flexure of the colon

The finding prompted a contrast-enhanced abdominal CT scan, which showed a large lesion infiltrating the entire right pararenal space with multisystem involvement (Figure 2).

**Figure 3:**
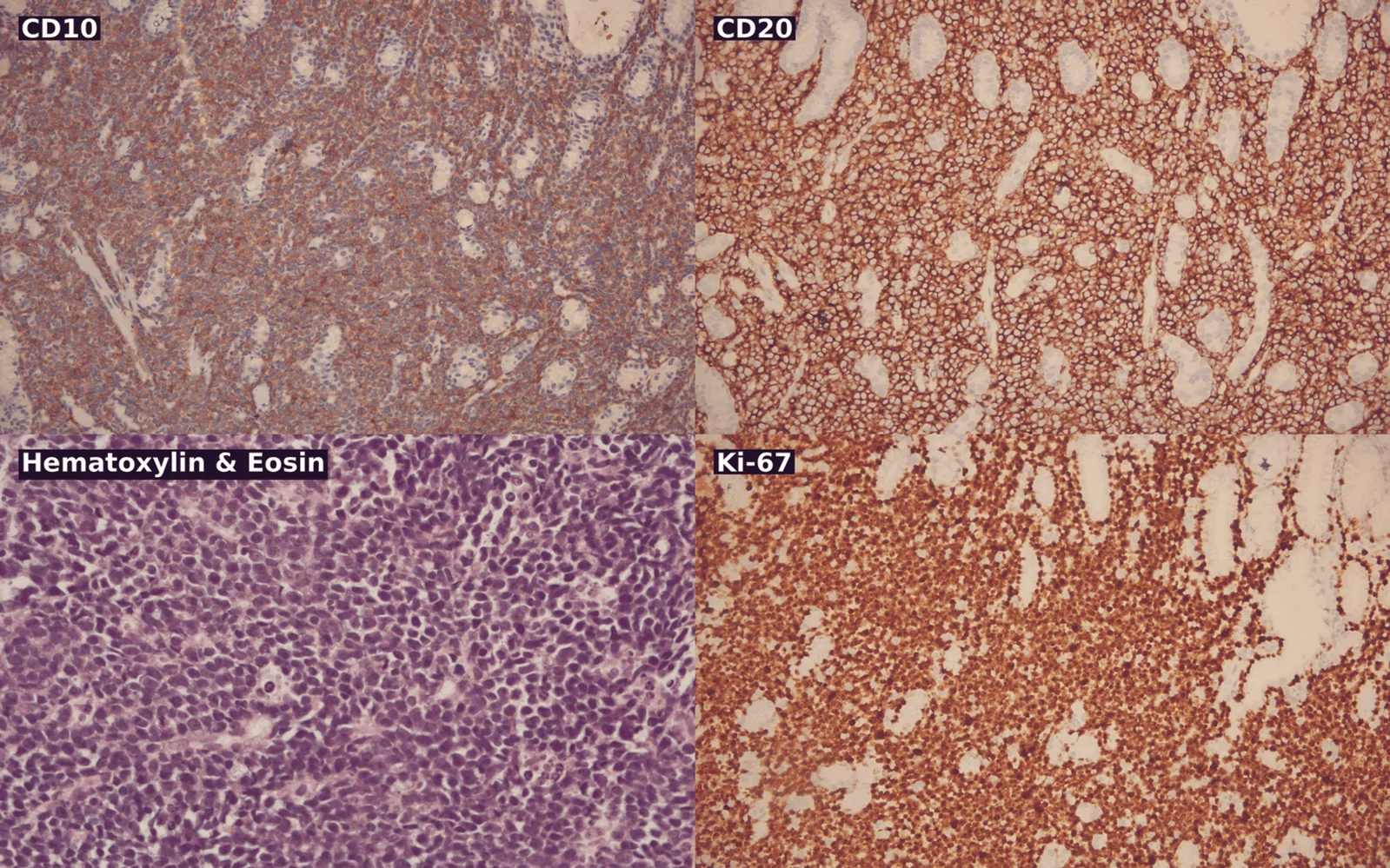
Histopathology of the retroperitoneal mass biopsy Intermediate-sized cells with nuclear molding and nuclear irregularity, associated with a high proliferative index. Immunohistochemistry shows positivity for CD20, CD10, MUM1, BCL2, and focal BCL6 expression, with a Ki-67 index of approximately 100%. The tumor cells are negative for CD30 and cyclin D1. Hematoxylin and eosin (H&E) was examined at 40× magnification, while CD10, CD20, and Ki-67 immunohistochemical stains were examined at 10× magnification.

Suspecting a lymphoproliferative syndrome, clinicians performed a percutaneous biopsy of the retroperitoneal lesion. Histopathological analysis revealed high-grade aggressive B-cell lymphoma with MYC mutation (Figure 3).

**Figure 4:**
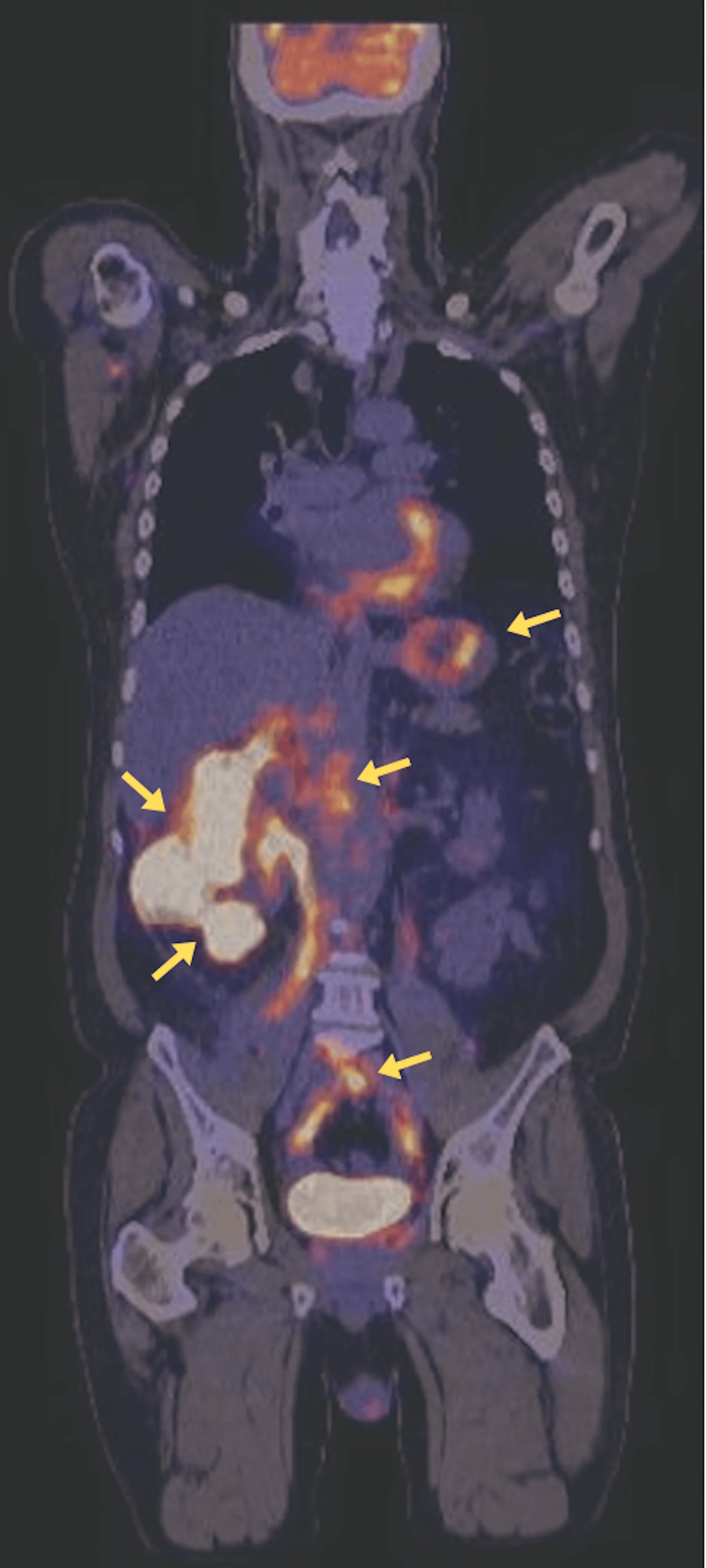
PET/CT with 18F-FDG Tumor viability due to lymphoma with nodal involvement above and below the diaphragm, as well as gastric, right crus, ipsilateral kidney, and mesorectal fascia involvement. PET/CT: positron emission tomography/computed tomography; 18F-FDG: fluorodeoxyglucose F-18

Before initiating treatment with the R-CHOP (cyclophosphamide, doxorubicin hydrochloride, and vincristine sulfate) regimen, a PET/CT scan was performed (Figure 4).

**Figure 5:**
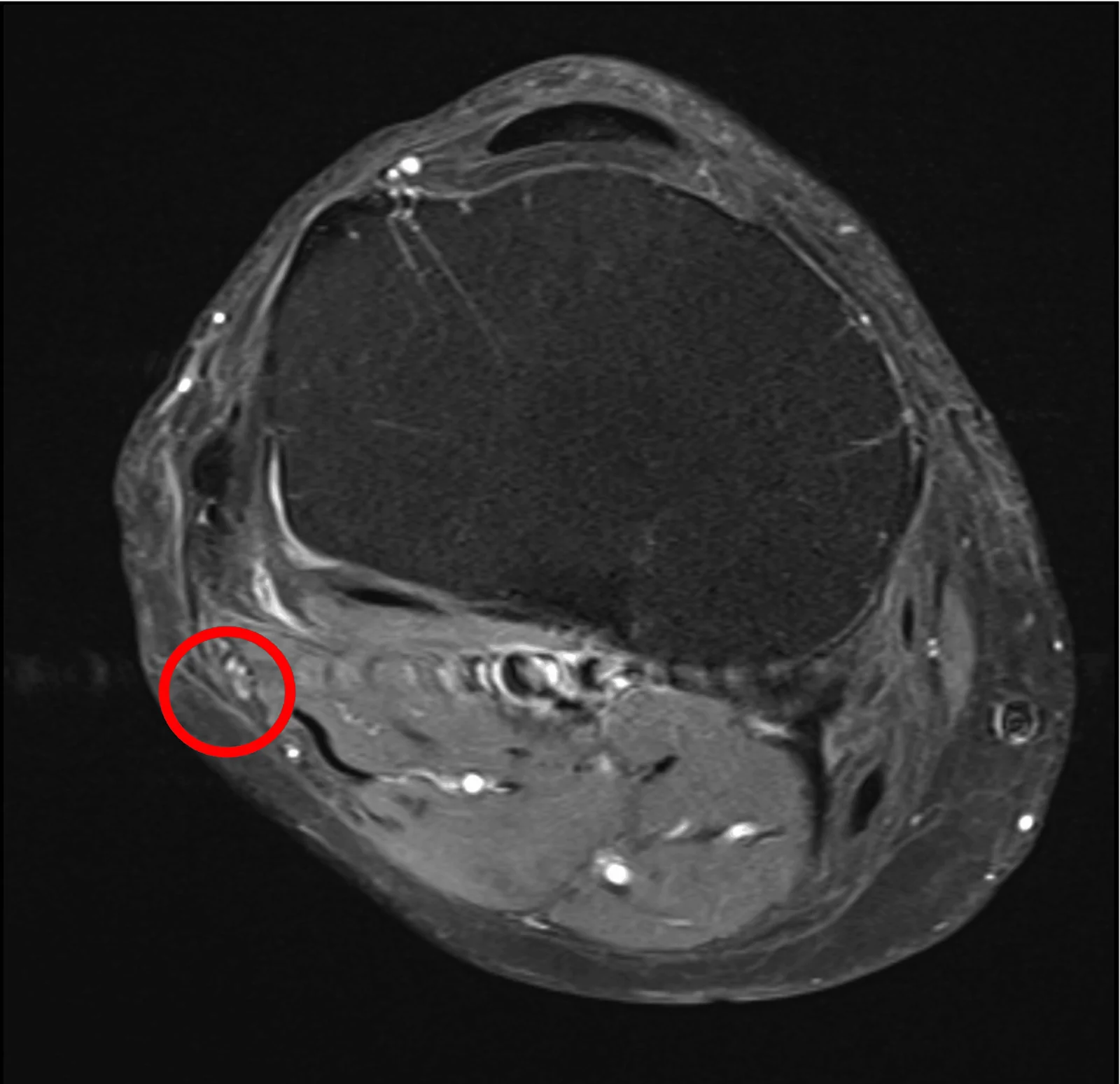
Right knee MRI Axial T1-weighted sequence with contrast and fat saturation—thickening of the peroneal nerve with heterogeneous enhancement of its fibers.

**Figure 6:**
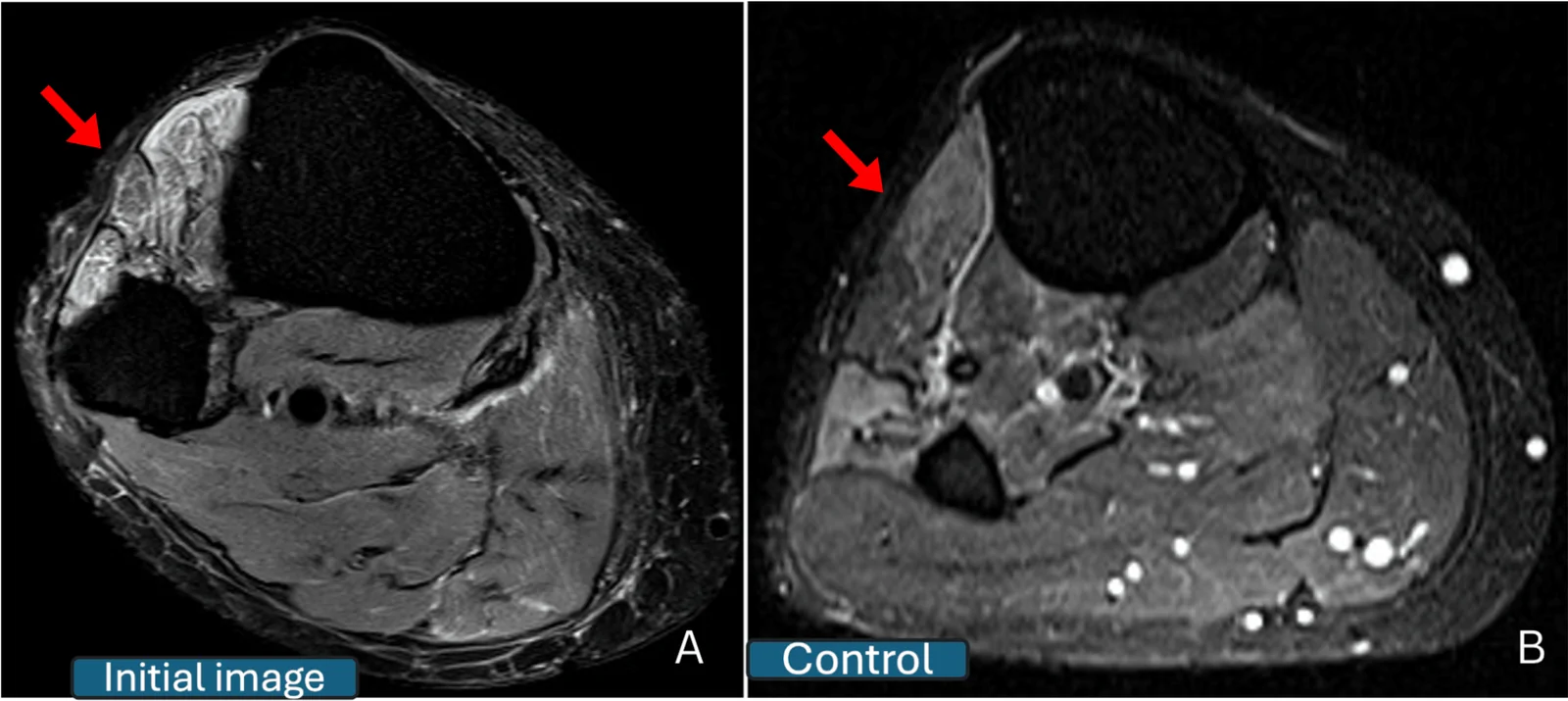
Serial axial MRI of the right leg demonstrating denervation changes A: Axial PD sequence with fat saturation. Corresponds to the initial study, showing muscle edema in the anterolateral compartment of the leg (including the peroneus longus, extensor digitorum longus, and tibialis anterior) due to acute denervation. B: Axial STIR sequence. Follow-up study demonstrating persistent mild muscle edema in the peroneus longus with marked atrophic changes, consistent with subacute/chronic denervation.

Upon stabilization, after further evaluation for the right foot drop, two primary clinical hypotheses were considered: compression of the peroneal nerve at the knee level secondary to cachexia and neurolymphomatosis. Repeat EMG indicated right peroneal nerve involvement, with conduction block at the level of the knee and secondary axonal injury. A contrast-enhanced knee MRI subsequently demonstrated involvement of the right peroneal nerve with an acute denervation pattern (Figures 5, 6A).

**Figure 7:**
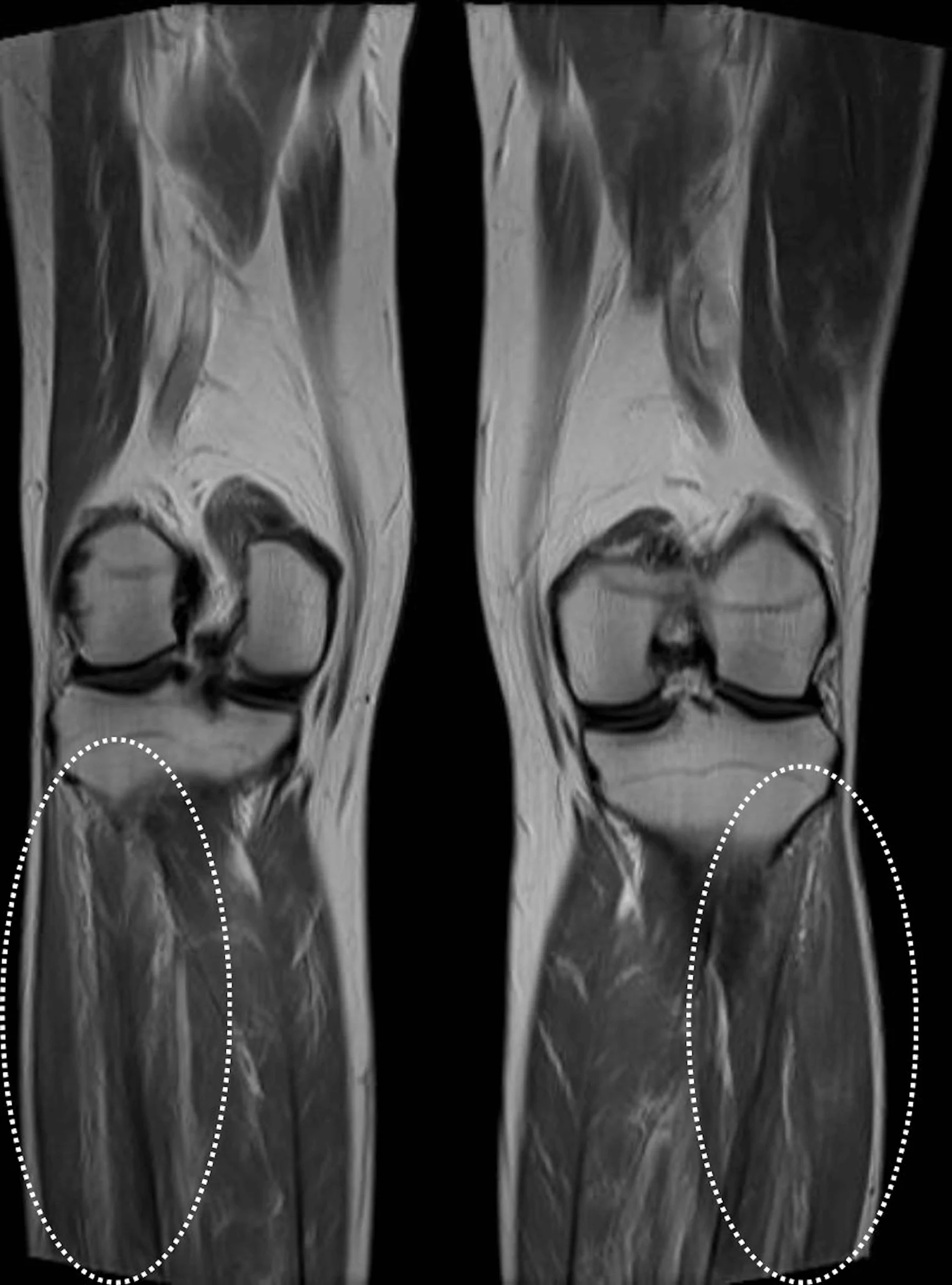
Coronal T1 sequence Coronal T1 sequence demonstrating atrophic changes in the anterolateral compartment of the right leg, with fatty infiltration and volume loss compared to the healthy (left) side.

The patient subsequently completed four cycles of R-CHOP chemotherapy, after which significant neurological and radiological improvement was documented. Clinically, a substantial motor recovery was achieved, with right foot dorsiflexion strength improving from MRC grade 2/5 (+) to a functional grade 4/5 (++/++), characterized by a partial but functional dorsiflexion. A follow-up contrast-enhanced MRI of the knee demonstrated a resolution of the previous abnormal nerve enhancement and a regression to a subacute denervation pattern in the anterolateral compartment of the leg (Figures 6B, 7, and Video 1).

**Video 1:** Follow-up contrast-enhanced MRI of the knee

After multidisciplinary assessment and in accordance with the established clinical criteria for paraneoplastic syndromes, the findings supported the diagnosis of paraneoplastic motor mononeuropathy secondary to lymphoma.

## Discussion

PNS are rare disorders that manifest in cancer patients without direct tumor invasion, compression, or metastatic seeding of the nervous system. These syndromes typically arise from an autoimmune response triggered by shared antigens between the underlying malignancy and neural structures [6,7]. In the context of NHL, NL, defined as the direct infiltration of malignant cells into the peripheral or cranial nerves, represents the differential diagnosis par excellence and poses a significant diagnostic challenge [7].

In our patient, imaging initially revealed a retroperitoneal mass, and histopathological analysis confirmed a high-grade aggressive B-cell lymphoma with a MYC mutation. Given the coexistence of an asymmetric motor neuropathy and lymphoma, NL was highly suspected. Furthermore, the initial MRI findings, which demonstrated thickening of the right peroneal nerve with patchy contrast enhancement and acute muscle denervation, are radiologically compatible with both NL and paraneoplastic neuropathy [7,8].

Despite these overlapping features, several clinical and therapeutic factors rendered direct lymphomatous infiltration significantly less probable. First, NL characteristically presents with severe, progressive, and intractable neuropathic pain; conversely, our patient exhibited a completely painless, purely motor deficit. Second, NL typically exhibits a poor response to conventional systemic chemotherapy alone, often requiring intrathecal therapy or targeted radiation due to the blood-nerve barrier, and carries an unfavorable prognosis. In this case, the patient demonstrated a dramatic and rapid clinical and radiological response after completing four cycles of the standard systemic R-CHOP regimen within a two-month interval. His right foot dorsiflexion strength significantly improved from MRC grade 2/5 (+) to functional grade 4/5 (++/++). Concurrently, follow-up MRI confirmed the complete resolution of abnormal nerve enhancement and a transition to a subacute denervation pattern, a trajectory highly atypical for active neurolymphomatosis.

We acknowledge certain limitations in our diagnostic workup, specifically the absence of a nerve biopsy and onconeural/surface antibody panels. A peripheral nerve biopsy was deemed unfeasible and clinically counterproductive; it is a highly invasive procedure that carries a substantial risk of inflicting permanent neurological deficits on an already compromised peroneal nerve, and its yields are frequently inconclusive. Regarding the omission of antibody testing, paraneoplastic syndromes secondary to NHL are notoriously seronegative or associated with unclassified, ultra-rare antibodies. Although onconeural and surface antibody studies are considered critical components in the evaluation of motor neuropathies, positive findings are particularly rare in the setting of lymphoma [9]; consequently, a negative antibody panel would not have ruled out a paraneoplastic etiology.

Ultimately, according to the 2004 Graus criteria updated in 2022, the clinical presentation and therapeutic behavior firmly establish the diagnosis. A non-classical neurological syndrome that significantly regresses or completely resolves following oncological treatment alone-in the strict absence of immunotherapy-fulfills the definitive criteria for a paraneoplastic syndrome [4,10]. This diagnostic conclusion remains robust throughout the patient's multi-month clinical follow-up, confirming a stable and sustained neurological recovery.

## Conclusions

We present a case of motor neuropathy in a patient with aggressive B-cell lymphoma. This report highlights the importance of neuropathies associated with NHL, which, although rare, are clinically significant. In this case, the diagnosis favored a paraneoplastic neuropathy over NL based on the clinical course, imaging findings, EMG, exclusion of other likely causes, and neurological improvement following oncologic treatment. However, definitive confirmation with nerve biopsy or onconeural antibody testing was not available. When facing similar circumstances, paraneoplastic mechanisms should be considered in the differential diagnosis of peripheral neuropathies associated with lymphoma, with asymmetric sensorimotor neuropathies being among the most common presentations. Neurological manifestations occurring in patients with underlying malignancies should be recognized, and PNS should be included in the differential diagnosis, even when the clinical and radiological findings overlap with NL. A multidisciplinary approach that includes imaging, EMG, clinical evaluation, and adequate follow-up is necessary for an early and accurate diagnosis.
